# Uterine Artery Pulsatility Index as a Pre-eclampsia Predictor in the 3 Trimesters in Women with Singleton Pregnancies

**DOI:** 10.1055/s-0041-1740273

**Published:** 2021-12-21

**Authors:** Yuly Natalia Guzmán, Montserrat Uriel, Alexandra Porras Ramírez, Ximena Carolina Romero

**Affiliations:** 1 Universidad El Bosque, El Bosque Research Group of Maternal Fetal Medicine and Gynecology, Bogotá, Colombia; 2 Universidad El Bosque, El Bosque Research Group of Maternal Fetal Medicine and Gynecology, Ecodiagnóstico El Bosque SAS, Los Cobos Medical Center, Bogotá, Colombia; 3 Universidad El Bosque, Research Group Community Medicine and Collective Health, Los Cobos Medical Center, Bogotá, Colombia; 4 Universidad El Bosque, El Bosque Research Group of Maternal Fetal Medicine and Gynecology, Ecodiagnóstico El Bosque SAS, Los Cobos Medical Center, Bogotá, Colombia

**Keywords:** uterine artery doppler, preeclampsia; pregnancy, perinatal care, screening, doppler da artéria uterina, pré-eclâmpsia; gravidez, cuidado perinatal, triagem

## Abstract

**Objective** To evaluate the mean uterine artery pulsatility index (UtAPI) in each trimester of pregnancy as a predictor of early or late pre-eclampsia (PE) in Colombian pregnant women.

**Methods** The UtAPI was measured in singleton pregnancies in each trimester. Uterine artery pulsatility index as predictor of PE was evaluated by odds ratio (OR), receiver operating characteristic (ROC) curves, and Kaplan-Meier diagram.

**Results** Analysis in the 1 ^st^ and 3 ^rd^ trimester showed that abnormal UtAPI was associated with early PE (OR: 5.99: 95% confidence interval [CI]: 1.64–21.13; and OR: 10.32; 95%CI: 2.75–42.49, respectively). Sensitivity and specificity were 71.4 and 79.6%, respectively, for developing PE (area under the curve [AUC]: 0.922). The Kaplan-Meier curve showed that a UtAPI of 0.76 (95%CI: 0.58–1.0) in the 1 ^st^ trimester was associated with early PE, and a UtAPI of 0.73 (95%CI: 0.55–0.97) in the 3 ^rd^ trimester was associated with late PE.

**Conclusion** Uterine arteries proved to be a useful predictor tool in the 1 ^st^ and 3 ^rd^ trimesters for early PE and in the 3 ^rd^ trimester for late PE in a pregnant population with high prevalence of PE.

## Resumo

**Objetivo** Avaliar o índice médio de pulsatilidade da artéria uterina (UtAPI) em cada trimestre da gravidez como preditor de pré-eclâmpsia (PE) precoce ou tardia em gestantes colombianas.

**Métodos** O UtAPI foi medido em gestações únicas em cada trimestre. O UtAPI como preditor de PE foi avaliado por odds ratio (OR), curvas receiver operating characteristic (ROC) e diagrama de Kaplan-Meier.

**Resultados** A análise no 1° e 3° trimestres mostrou que um UtAPI anormal foi associado com PE inicial (OR: 5,99; intervalo de confiança [IC] 95%: 1,64–21,13; OR: 10,32; IC95%: 2,75–42,49, respectivamente). A sensibilidade e a especificidade foram de 71,4 e 79,6%, respectivamente, para o desenvolvimento de PE (area under the curve [AUC]: 0,922). A curva de Kaplan-Meier mostrou que um UtAPI de 0,76 (IC95%: 0,58–1,0) no 1° trimestre foi associado com PE precoce, e que um UtAPI de 0,73 (IC95%: 0,55–0,97) no 3° trimestre foi associado com PE tardia.

**Conclusão** As artérias uterinas mostraram ser uma ferramenta preditora útil no 1° e 3° trimestres para PE inicial e no 3° trimestre para PE tardia em uma população de gestantes com alta prevalência de PE.

## Introduction

Pre-eclampsia (PE) is a disease with a high percentage of maternal and perinatal complications and continues to be one of the main causes of maternal death worldwide. 1 2 3 4 Several methods have been studied to classify low- and high-risk populations; in recent years, early screening has been increasingly performed in an attempt to reduce PE prevalence through specific pharmacological interventions. 4 5 6

Several research groups, such as the National Institute for Health and Care Excellence (NICE), the American College of Obstetricians and Gynecologists (ACOG), and the Fetal Medicine Foundation (FMF), have analyzed screening methods for PE in which different parameters regarding maternal history, clinical symptoms, laboratory results, and biophysical variables are evaluated. One of the biophysical measures is the mean uterine artery pulsatility index (UtAPI). 7 8

The pathophysiology of PE has been explained by several theories. The most accepted etiology in recent years is that PE is a pathology secondary to an altered placental development, manifested by the increase in the flow resistance of the uterine arteries. 1 8 9 10 11 These alterations have been observed in the 1 ^st^ , 2 ^nd^ , and 3 ^rd^ trimesters of the pregnancies that develop PE. 12 13 Furthermore, uterine arteries were compared using Doppler in each trimester for predicting late PE; as a result, late PE can be best predicted during the 3 ^rd^ trimester. 13 14 A systematic review has described that the 2 ^nd^ trimester is the best time to predict overall PE. 15 Moreover, relevant international studies have evaluated the distribution of UtAPI in different populations during the course of pregnancy. 1 7 16 17 18 In Latin America, there are no comparable studies that provide information about these values throughout the pregnancy or their association with PE.

Pre-eclampsia is classified as early and late, being differentiated by gestational age at onset. In early PE cases, the presentation is before 34 gestational weeks (GWs). 10 19 This period is frequently associated with various unfortunate events, such as severe PE, eclampsia, hemolysis, elevated liver enzymes, and low platelet count (HELLP) syndrome, maternal death, admission to obstetric intensive care unit (ICU), admission to neonatal ICU, low birthweight, and perinatal death. 3

According to information dated from 2014, hypertensive disorders are the second leading cause of death in pregnant women in the Latin American and Caribbean population. 2 20 In Bogotá, Colombia, PE is the leading cause of maternal death, according to the Department of National Statistics – Data Bases of 2017. 21 Thus, early screening for hypertensive disorder is crucial to optimize or develop clinical strategies and public interest to reduce PE cases. Unfortunately, in the Colombian population, no information about specific characteristics of uterine artery Doppler study during the three trimesters of pregnancy is available.

The present study aimed mainly to evaluate the mean UtAPI measured in each trimester of pregnancy as a predictor of early or late PE in a sample of Colombian pregnant women.

## Methods

The data for the present study were derived from a database of 566 pregnant women > 14 years old with singleton pregnancies assessed 2 two hospitals in the city of Bogotá, Colombia, from a prospective study in which predictive tests of PE were analyzed in Colombian pregnant women in the period between October 2014 and March 2018. For the present work, pregnancies with major fetal malformations, chromosomopathies, and those under threat of miscarriage were excluded. During the 1 ^st^ visit, between 11 + 0 and 13 + 6 GWs, a clinical history regarding the sociodemographic and clinical characteristics of the patients was obtained. Doppler study of the uterine arteries was performed in the 1 ^st^ trimester between 11 + 0 and 13 + 6 GWs, in the 2 ^nd^ trimester between 18 + 0 and 24 + 0 GWs, and in the 3 ^rd^ trimester between 28 + 0 to 34 + 0 GWs. Gestational age was determined by measuring the fetal crown–rump length from 11 + 0 GWs to 13 + 0 GWs.

Regarding the characteristics of the patients, the following demographic variables are included: maternal age, ethnic origin, and socioeconomic level (classified as high, middle, and low). The following clinical variables were also considered: medical history of chronic hypertension, pregestational diabetes, obesity defined as body mass index (BMI) ≥ 30 Kg/m2, systemic lupus erythematosus, antiphospholipid antibody syndrome, personal and family history of PE, parity (nulliparity versus non-nulliparity), smoking habit during pregnancy, maternal BMI, gestational age at the time of PE diagnosis, and gestational age at the end of pregnancy.

Doppler ultrasound examinations were performed transabdominally. Uterine artery pulsatility index measuring techniques were used according to the guidelines by Khalil et al. 22 . At 11 + 0 to 13 + 6 GWs, a midsagittal section of the uterus was obtained, and the cervical canal and internal cervical os were identified. Subsequently, the transducer was gently tilted from side to side, and color flow mapping was used to identify each uterine artery measurements were taken before the uterine artery branches into the arcuate arteries. 22 23 24

At 18 + 0 to 24 + 6 GWs and at 28 + 0 to 34 + 0 GWs, the transducer was placed longitudinally in the iliac fossa, parallel to the iliac crest and to the uterine wall. Then, color Doppler was used to identify each uterine artery at the apparent crossover with the external iliac arteries. After the identification of each uterine artery, pulsed-wave Doppler was used, with the sampling gate set at between 2 and 3 mm to cover the whole vessel. Care was taken to ensure that the angle of insonation was *<* 30° and that the peak systolic velocity was *>* 60 cm/s, so that the uterine artery, rather than the arcuate artery, was examined. When three similar waveforms were obtained consecutively, the pulsatility index was measured and the mean UtAPI of the left and right arteries was calculated. 22 23 The presence or absence of a diastolic notch was not considered in the present study. The mean UtAPI was analyzed qualitatively according to the normality and abnormality criteria described by Gómez et al. 17 in their study, which was developed in a Spanish population, using the 95 ^th^ percentile; hence, UtAPI values above this range were considered abnormal. All Doppler studies were performed by sonographers who had received the Certificate of Competence in Doppler of the FMF.

Patients were diagnosed with PE according to the 2013 diagnostic criteria of hypertension in pregnancy. 25 26 27 28 In addition, the gestational age at the onset of PE was considered; PE was classified as early PE when it starts before 34 GWs and as late PE when it starts after this gestational age. 10 19

Informed consent was obtained from all patients. The present study was approved by the ethical committees of each participating hospital. In addition, the ethical principles for human research from the Helsinki Declaration and the Colombian resolution 8430 of 1993 were considered in the present study, and it was classified as an investigation with minimum risk. 29

Absolute and relative frequencies were performed with their respective confidence intervals (CIs) to describe the characteristics of the patients. Comparisons of groups were analyzed by the student t-test, the Mann-Whitney U test, and the Fisher test, considering *p* < 0.05 to stablish statistical differences. To find the possible associations between uterine pulsatility in each trimester and the development of PE, the odds ratio (OR) was calculated. Receiver operating characteristic (ROC) curves and the area under the curve (AUC) were made to calculate the sensitivity and specificity of UtAPI in order to determine the risk of developing PE. Kaplan-Meier probability diagrams were calculated to evaluate the probability of developing PE (early and late) related with gestational age in the upper and lower quintile of the UtAPI.

## Results

The present study included 527 pregnant women who met the inclusion criteria, out of a total of 566 women who were studied. The average age was 27.4 years old, and 97.3% of the participants were of mixed race. Furthermore, 96.9% of these women were from middle and low socioeconomic levels. Sociodemographic characteristics and pathological history of the study population are summarized in Table 1 , as well as the comparation between groups.

**Table 1:** Sociodemographic and clinical characteristics

Sociodemographic characteristics/ pathological history	Pregnant women included ( *n* = 527)	Without PE ( *n* = 485)	Early PE ( *n* = 8)	Late PE ( *n* = 34)	*p-value*
Median age, years old median (IQR)	27.0 (14–44)	27.8 (14–44)	27.9 (17–38)	30.4 (15–42)	0.062*
BMI in Kg/m ^2^ (IQR)	24.6 (16.7–40.5)	26.6 (17.9–400)	29.0 (18.5–39.8)	28.3 (19.3–35.5)	0.429**
Gestational age at delivery in weeks (IQR)	39.0 (28.2–43.1)	37.5 (29.3–43.1)	35.5 (30.4–40.4)	37.5 (36–40.4)	0.721**
Obesity %	47 (8.9)	39 (8.0)	2 (25.0)	6 (17.6)	0.043***
Pregestational diabetes %	3 (0.56)	2 (0.4)	1 (12.5)	0	–
CH %	14 (2.6)	6 (1.2)	5 (62.5)	3 (8.8)	0.399***
SLE %	2 (0.38)	1 (0.2)	0	1 (2.9)	–
APS %	1 (0.19)	0	1 (12.5)	0	–
Smoking habit %	47 (8.9)	40 (8.2)	3 (37.5)	4 (11.7)	0.04***
Nulliparity %	203 (38.5)	191 (39.3)	3 (37.5)	9 (26.4)	0.002***

Abbreviation: APS, antiphospholipid antibody syndrome; BMI, body mass index; CH, chronic hypertension; IQR, interquartile range; PE, pre-eclampsia; SLE, systemic lupus erythematosus.

* Mann-Whitney U test; **Student's t-test; *** Fisher test.

In the present study, 42 women (7.9%) had PE, of whom 34 (6.4%) had late PE and 8 (1.5%) had early PE. The average gestational age at the moment of diagnosis of early and late PE was 30 + 1 GWs (21 + 0 to 33 + 0 GWs) and 37 + 1 GWs (34 + 0 to 40 + 4 GWs), respectively. In the 1 ^st^ trimester, 12.9% (68) of the pregnant women had an UtAPI > 95 ^th^ percentile. The mayor ratio of patients with UtAPI>95 ^th^ percentile in the 1st trimester was obtained in the group of patients who developed early PE, 5 patients of the 8 (62.5%). In the 2 ^nd^ trimester, 14 women did not attend the ultrasound appointment, but, of the new total of patients who attended to the 2 ^nd^ appointment, 9.6% (51) had abnormal UtAPI. Besides, the group that developed early PE showed the highest ratio of patients with UtAPI > 95 ^th^ percentile (5 patients of the 8 who developed early PE). Finally, in the 3 ^rd^ trimester, 20 women were lost to follow-up. Of the patients evaluated in the 3 ^rd^ trimester, 12.7% (67) of women who were in the 3 ^rd^ trimester of gestation had a UtAPI > 95th percentile; the highest percentage of patients with UtAPI > 95 ^th^ percentile was in the group of early PE (75%) (6 patients of the 8 that developed early PE). The analysis of the ORs showed a positive association between abnormal UtAPI in the 1 ^st^ trimester and the development of early PE (OR: 5.9; 95%CI: 1.64–1.13), with statistically significant differences. An association between having UtAPI > 95 ^th^ percentile in the 3 ^rd^ trimester and early PE (OR: 10.32; 95%CI: 2.75-42.49) was found, as shown in Table 2 .

**Table 2:** Bivariate analysis (OR) of abnormal UtAPI and developing or not PE

	Without PE	Early PE	Late PE
UtAPI > 95 ^th^ percentile by trimester	**OR (95%CI)**	**OR (95%CI)**	**OR (95%CI)**
First	1.01 (0.98–1.23)	5.99(1.64–21.13)*	0.46 (0.07–1.73)
Second	0.98 (0.75–2.36)	4.74 (0.94–19.66)	1.98 (0.64–5.21)
Third	1.10 (0.45–1.87)	10.32 (2.75–42.49)*	1.65 (0.59–4.05)

Abbreviations: CI, confidence interval; OR, odds ratio; PE, pre-eclampsia; UtAPI, uterine artery pulsatility index.

* Statistically significant between groups.

**Fig. 1:**
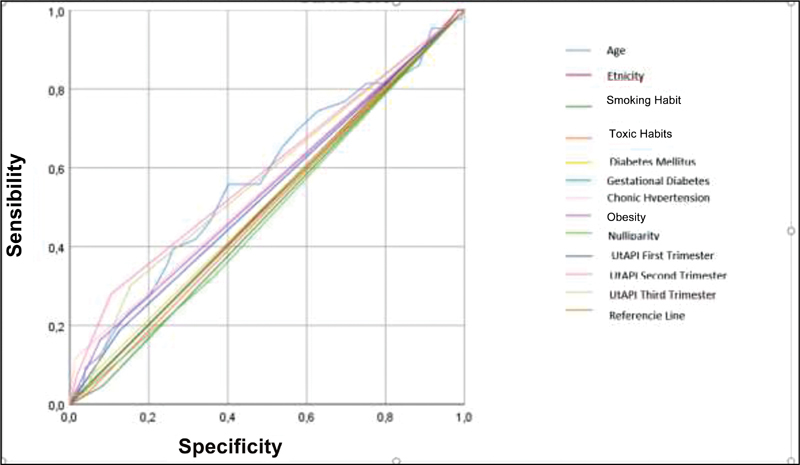
Receiver operating characteristic curve.

Receiver operating characteristic curves were made to evaluate the ability to discriminate the likelihood of developing PE. The ROC shows the importance of the UtAPI in the 1 ^st^ and the 2 ^nd^ trimester, so that the model achieves a prediction of 92.2% of the risk of developing PE, with a sensitivity and a specificity of 71.4 and 79.6%, respectively, by using all the characteristics described in Figure 1 . At the 1st and 2nd trimesters, the sensitivity of the UtAPI was 20% of each one.

**Fig. 2:**
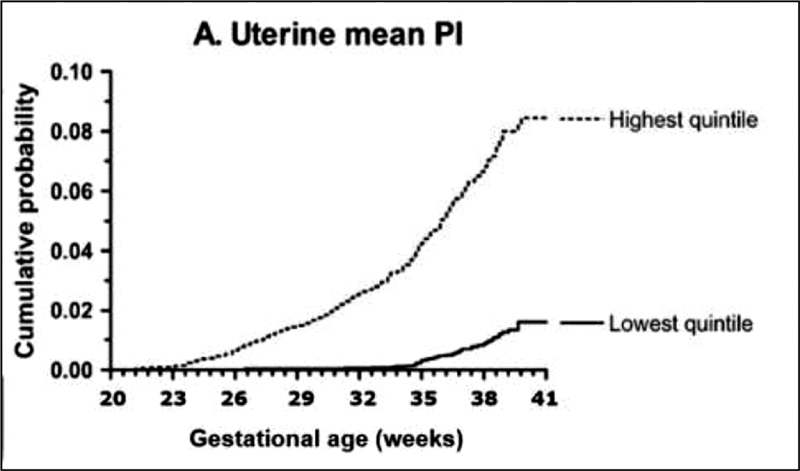
Kaplan–Meier probability diagram of early and late pre-eclampsia and their relationship with gestational age and the upper and lower quintiles of uterine artery pulsatility index.

The analysis of the Kaplan-Meier probability diagram demonstrated the relationship between the upper quintile of the UtAPI 0.76 (95%CI: 0.58–1.0) in the 1 ^st^ trimester and the development of early PE, with statistically significant results. The risk of late PE for the upper quintile of UtAPI 0.73 (95%CI: 0.55–0.97) in the 3 ^rd^ trimester was statistically significant. The Kaplan-Meier cumulative risk of PE is shown in Figure 2 .

## Discussion

According to the findings of the present, PE was found in 7.9% of the participants, suggesting that the Colombian population has a higher incidence of PE than the world population (between 5 and 7%), with a high percentage of maternal and perinatal complications. 30 31 32 Additionally, in relation to PE onset, 34 (6.4%) pregnant women had late PE and 8 (1.5%) had early PE onset.

Considering the elevated incidence of this hypertensive disease, it is important to have knowledge about its probable etiology. The increase in the flow resistance of the uterine arteries is one of the bases of the pathophysiology of PE, as referenced by O'Gorman et al. 12 These alterations could be shown by Doppler changes in uterine arteries in each trimester or in all trimesters; consequently these variations have been observed in the 1 ^st^ , 7 the 2 ^nd^ , 33 and the 3 ^rd^ trimesters. 11 33 Other researches, as Mula et al., 13 have examined Doppler changes of the uterine artery in all trimesters, as our study, in which women were evaluated by trimester attending their routine care. In the present study, abnormal UtAPI was found in all 3 trimesters of pregnancy in 16.6% of the pregnant women with PE.

In this context, Doppler changes found in uterine arteries by trimester are consistent with other research. In 2016, Arrue et al. 34 concluded that patients with an abnormal UtAPI in the 3 ^rd^ trimester had a higher rate of PE and adverse perinatal outcomes independent of UtAPI values in the 1 ^st^ and 2 ^nd^ trimesters of pregnancy. In 2013, Jamal et al. 33 found that an elevated UtAPI in the 2 ^nd^ and 3 ^rd^ trimesters of pregnancy was associated with a higher risk of adverse pregnancy outcomes, such as PE, intrauterine growth restriction (IUGR), intrauterine fetal death, and preterm delivery, than in the 1 ^st^ trimester. Notably, in the present study, abnormal UtAPI in the 1 ^st^ and 3 ^rd^ trimesters was associated with early PE, with a statistically significant difference. The early PE association in the 3 ^rd^ trimester was higher than in the 1 ^st^ trimester, conforming to the abovementioned studies in which an abnormal UtAPI in the 3 ^rd^ trimester had a greater risk of developing adverse outcomes, such as PE.

Likewise, regarding the 2 ^nd^ trimester, a systematic review and meta-analysis by Cnossen et al. 15 has found a higher prediction of PE and IUGR when UtAPI was > 95 ^th^ percentile alone or combined with notching in the 2 ^nd^ trimester when compared with abnormal UtAPI in 1 ^st^ and 3 ^rd^ trimesters. In 2013, 2,410 pregnant women were evaluated to determine the biological interaction between uterine artery Doppler and blood pressure in the 2 ^nd^ trimester for the development of early and late PE. 35 In this study, Takahashi et al. 35 found a cumulative risk of early PE in women with both abnormal UtAPI and elevated blood pressure, which is in contrast with the present study, in which the association was not statistically significant in the 2 ^nd^ trimester.

Additionally, in 2019, Mula et al. 13 evaluated the prediction of late PE by measuring the UtAPI in each trimester; they found that UtAPI measurement in the 3 ^rd^ trimester was more sensitive (78%) and specific (82%) than in the 2 other trimesters (AUC: 0.86). In the present study, according to ROC curves (AUC: 0.922), the sensitivity and specificity to determine the risk of developing PE were 71.4 and 79.6%, respectively.

Focusing on PE predictor tools, similarly, screening methods for PE with evaluation of different parameters, such as maternal history, clinical, laboratories, and biophysical variables (such as UtAPI, evaluated in the present study), were analyzed. Andrietti et al., 14 who demonstrated that the combined measurement of UtAPI, mean arterial pressure (MAP), and serum placental growth factor in the 1 ^st^ and/or 2 ^nd^ trimesters did not improve the prediction of early PE compared with screening in the 3 ^rd^ trimester. In the present study, more cases of late PE were identified by UtAPI measurements in the 3 ^rd^ trimester. 14 In general, among the biophysical measures, UtAPI has become one of the most useful parameters for PE screening since it is more available than measurements of placental proteins, which are not accessible for the entire population as part of their health insurance plan, as it happens in Colombia.

Consequently, UtAPI measurement has been evaluated with inconsistent results between researches, with the present study being one of the few that analyzed the prognostic ability in each trimester of pregnancy in the same group of women, and these results show in which trimester the measurement of uterine arteries was more efficient to improve the PE screening rate in a country with a high incidence of PE. The main limitations of the present study were that we incurred a random error because of the small sample size. Furthermore, a selection bias was observed, given that the patients included in the present study corresponded to a specific population of Bogotá.

## Conclusion

In conclusion, the measurement of the UtAPI in the 1 ^st^ and 3 ^rd^ trimesters of pregnancy may be a useful technique for the screening of early PE and, in 3 ^rd^ trimester, for late PE. However, in the present study, the result of this predictor tool was limited by the sample size. Hence, further studies are necessary to validate the predictive and prognostic ability of UtAPI in each trimester regarding PE in the Colombian population in isolation and in combination with other PE predictors.
